# THE MOVING FORWARD NURSING HOME COALITION: A CALL TO ACTION TO SUPPORT NURSING HOME REFORM

**DOI:** 10.1093/geroni/igad104.0963

**Published:** 2023-12-21

## Abstract

The Moving Forward Nursing Home Quality Coalition advances the goals outlined by the 2022 National Academies of Sciences, Engineering, and Medicine (NASEM) report aimed at improving nursing home (NH) quality. Over two years, Coalition committee members – along with a network of national leaders, nursing home residents, and the general public – will develop, test and promote action plans that will improve the way the United States finances, delivers, and regulates care in Nursing Homes. The Coalition is comprised of a steering committee and working committees that align with and advance the seven NASEM goals including; 1) advancing delivery of person-centered care; 2) ensuring a well-prepared and compensated workforce; 3) increasing financial transparency and accountability; 4) creating a rational and robust financing system; 5) designing more effective and responsive quality assurance; 6) expanding quality measurement and continuous quality improvement; and 7) adopting health information technology. Coalition committees have engaged in three of four phases of their two-year work plan including convening stakeholders to prioritize and develop action plans to test and promote change. Throughout the work, committee collaboration has ensured action plans are refined and expanded in synergistic ways to shape state and national policy. This presentation will provide an overview of the broader Coalition work and give context for the other presentations which provide an example of collaborative work leading to proposed recommendations for critical policy and regulatory change around person-centered care planning.

